# Editorial: “Next-Generation Sequencing in Rare Genetic Diseases”

**DOI:** 10.3390/genes17101236

**Published:** 2026-10-08

**Authors:** Edoardo Errichiello, Paola Dimartino

**Affiliations:** 1 Unit of Medical Genetics, Department of Molecular Medicine, University of Pavia, 27100 Pavia, Italy; 2 Neurogenetics Research Center, IRCCS Mondino Foundation, 27100 Pavia, Italy

Rare genetic diseases (RGDs) affect more than 300–400 million people worldwide. For a substantial proportion of affected individuals, the absence of a definitive diagnosis leads to the so-called “diagnostic odyssey”. Achieving an accurate diagnosis of an RGD can improve clinical management, facilitate the identification of potential therapeutics, and help avoid unnecessary treatments that may be associated with non-negligible side effects. Over the last decade, the advent of next-generation sequencing (NGS) and the rapid development of omics sciences have profoundly revolutionized the diagnostic approach to RGDs. In this evolving landscape, bioinformatics tools and analytical strategies have likewise advanced considerably, in parallel with emerging technological approaches, such as long-read sequencing.

This Special Issue aims to highlight the contribution of these novel approaches to elucidating pathogenetic mechanisms, identifying novel genotype–phenotype associations, and characterizing the genetic architecture underlying RGDs, ultimately paving the way for the development of innovative therapeutic strategies.

In their study, Iovino et al. highlight an important yet sometimes overlooked aspect of NGS data: coverage uniformity. Reduced sequencing depth and batch-specific issues during library preparation can result in randomly distributed regions of low coverage. Importantly, such regions can also occur in whole-genome sequencing (WGS), challenging the common assumption that genome-wide coverage is inherently uniform. The authors show that even experiments otherwise considered to be of good quality may contain low-coverage regions encompassing disease-associated genes, potentially hindering the detection of pathogenic variants.

Carstens et al. provide a timely review of an emerging area of precision medicine, highlighting how the rapid identification of pathogenic variants through standardized, high-resolution NGS approaches is paving the way for increasingly tailored therapeutic strategies, including patient-specific RNA therapeutics, such as antisense oligonucleotides (ASOs).

Again, in the context of precision medicine, Innamorato et al. provide a compelling demonstration of how the combination of two innovative approaches, cell-free DNA sequencing (cfDNA-NGS) and long-read whole-genome sequencing (LR-WGS), can help resolve cases that remain unsolved using conventional diagnostic approaches. By leveraging the complementary strengths of these technologies, the authors were able to fully elucidate a complex case, ultimately enabling the implementation of an appropriate treatment and surveillance strategy for the patient.

Two papers in this issue, by Chałupczyńska and Theodosiou, focusing on Polish and Cypriot cohorts, respectively, underscore the importance of population-specific reference databases and bioinformatic pipelines that account for substantial local genetic variation to improve the filtering and classification of variants.

Lapunzina-Soler et al., studying a cohort of individuals with autosomal recessive polycystic kidney disease (ARPKD), further emphasize the critical importance of accurate variant phasing and of bioinformatic pipelines capable of reliably determining variant phase. This is particularly relevant for genes characterized by extensive allelic heterogeneity, such as *PKHD1*, where accurate phasing is essential to ensure a reliable molecular diagnosis.

Finally, the study by Carrer et al. underscores the value of blinded reanalysis of prenatal exome sequencing (pES) data, demonstrating that revisiting genomic findings in the context of evolving prenatal phenotypes can provide additional diagnostic insight. Their findings further reinforce that a negative pES result does not rule out the presence of an underlying syndromic disorder.

## Data Availability

No new data were created in this editorial.

